# Sorted Golden-step phase encoding: an improved Golden-step imaging technique for cardiac and respiratory self-gated cine cardiovascular magnetic resonance imaging

**DOI:** 10.1186/s12968-019-0533-8

**Published:** 2019-04-18

**Authors:** Liheng Guo, Daniel A. Herzka

**Affiliations:** Department of Biomedical Engineering, Johns Hopkins University School of Medicine, 720 Rutland Ave, Suite 726 Ross Building, Baltimore, MD 21205 USA

**Keywords:** Golden step, Self-navigation, Self-gating, Motion tracking, Pseudo-projections, Cine imaging, Dark flow artifacts

## Abstract

**Background:**

Numerous self-gated cardiac imaging techniques have been reported in the literature. Most can track either cardiac or respiratory motion, and many incur some overhead to imaging data acquisition. We previously described a Cartesian cine imaging technique, pseudo-projection motion tracking with golden-step phase encoding, capable of tracking both cardiac and respiratory motion at no cost to imaging data acquisition. In this work, we describe improvements to the technique by dramatically reducing its vulnerability to eddy current and flow artifacts and demonstrating its effectiveness in expanded cardiovascular applications.

**Methods:**

As with our previous golden-step technique, the Cartesian phase encodes over time were arranged based on the integer golden step, and readouts near *k*_*y*_ = 0 (pseudo-projections) were used to derive motion. In this work, however, the readouts were divided into equal and consecutive temporal segments, within which the readouts were sorted according to *k*_*y*_. The sorting reduces the phase encode jump between consecutive readouts while maintaining the pseudo-randomness of *k*_*y*_ to sample both cardiac and respiratory motion without comprising the ability to retrospectively set the temporal resolution of the original technique. On human volunteers, free-breathing, electrocardiographic (ECG)-free cine scans were acquired for all slices of the short axis stack and the 4-chamber view of the long axis. Retrospectively, cardiac motion and respiratory motion were automatically extracted from the pseudo-projections to guide cine reconstruction. The resultant image quality in terms of sharpness and cardiac functional metrics was compared against breath-hold ECG-gated reference cines.

**Results:**

With sorting, motion tracking of both cardiac and respiratory motion was effective for all slices orientations imaged, and artifact occurrence due to eddy current and flow was efficiently eliminated. The image sharpness derived from the self-gated cines was found to be comparable to the reference cines (mean difference less than 0.05 mm^− 1^ for short-axis images and 0.075 mm^− 1^ for long-axis images), and the functional metrics (mean difference < 4 ml) were found not to be statistically different from those from the reference.

**Conclusions:**

This technique dramatically reduced the eddy current and flow artifacts while preserving the ability of cost-free motion tracking and the flexibility of choosing arbitrary navigator zone width, number of cardiac phases, and duration of scanning. With the restriction of the artifacts removed, the Cartesian golden-step cine imaging can now be applied to cardiac imaging slices of more diverse orientation and anatomy at greater reliability.

**Electronic supplementary material:**

The online version of this article (10.1186/s12968-019-0533-8) contains supplementary material, which is available to authorized users.

## Background

Cardiovascular magnetic resonance (CMR) has to detect and account for the inevitable presence of cardiac and respiratory motion in order to avoid severe degradation of image quality. Given the complexity and limitations of using auxiliary devices to track cardiac [1–7] and respiratory [8–12] motion, a large number techniques have been reported in the literature to use CMR-measured signals to detect cardiac and/or respiratory motion, without the use of additional hardware. Termed “self-gated” or “self-navigated” imaging, this class of techniques extract various forms of motion information during imaging, with various precisions and at different costs to the acquisition of imaging data.

For example, some of the earliest self-gated imaging techniques sample the center of k-space at essentially no cost to imaging, enabling cine imaging, but because the k-space origin provides the coarsest depiction of motion (the average of the entire imaging slice or volume), it is useful only in limited scenarios [13–17]. Techniques on the other end of the precision-cost spectrum use real-time low-resolution 2D images [18–22] or localized tissue excitation [23, 24] to derive motion, enabling highly specific motion tracking, but they sample too slowly for cardiac motion tracking and interrupts acquisition too long for cine imaging because each motion measurement costs 10s to 100 s of milliseconds to execute.

Motion-tracking using 1D CMR readouts offers a more practical tradeoff between precision and cost. A linear readout acquired through the k-space origin corresponds to the 1D projection of the imaging slice or volume and provides more information than the k-space origin but costs much less time (few milliseconds) than 2D images or 2D excitation. In radial sequences, where each imaging readout is itself a projection, motion tracking is free of cost to imaging [15], but individual radial projections are not used for motion tracking in the literature presumably because tracking moving anatomies from a constantly changing projection angle is infeasible. In Cartesian sequences, the projection readout can be built into the routine traversal of k-space center in every repetition time (TR) [25–27] with sub-millisecond cost – fast enough to capture the cardiac motion, although developing such specialized sequences requires extensive gradient waveform design. Alternatively, one may obtain a projection by simply acquiring a Cartesian readout without phase encoding (PE). However, because such a readout costs several milliseconds and results in a gap in imaging, it is typically executed only at an interval of 10s of milliseconds [28–32]. Due to the regular disruption to imaging and the relatively low motion-sampling rate, cardiac cine imaging is not practical and usually only the slower respiratory motion is tracked.

Given that existing techniques can only track either cardiac or respiratory motion and do so with various overhead to imaging, we previously developed a self-gated 2D Cartesian cine imaging technique capable of tracking both cardiac motion and respiratory motion at no cost to imaging data acquisition [33]. In that technique, a small central portion (e.g. 5% or 10%) of the Cartesian *k*_*y*_ grid was designated as the “navigator zone,” and normal imaging readouts within this region were considered sufficiently close to *k*_*y*_ = 0 to be deemed “pseudo-projections,” from which both cardiac motion and respiratory motion were derived and used for self-gated cine reconstruction. During a scan, Cartesian readouts were continuously acquired, and a golden-step (GS) scheme was used to order their PEs – this key feature ensured that at any width of the navigator zone and any scan duration, imaging readouts would pseudorandomly fall within the navigator zone at approximately uniform intervals in both time and *k*_*y*_. As a result, minimal motion-related pre-planning was required because the zone width could be selected retrospectively and so could the number of cardiac phases. Because all cardiac phases were equally likely to receive data in any portion of the scan, the scan duration could also be arbitrary, i.e. it would not need to be planned with consideration to the number of cardiac phases or the segmentation scheme of k-space. Since the introduction of the original golden-angle MRI [34], there have been numerous reports of golden ratio-inspired imaging techniques such as Tiny Golden Angle [35, 36], Pseudo-Golden Angle [37, 38], and other similar pseudo-random acquisition schemes [39–41]. While they applied to radial imaging and did not deal with motion, our GS work was the first in Cartesian imaging that was also cable of cardiac and respiratory dual motion tracking.

However, because our previous GS scheme used a balanced steady-state free precession (bSSFP) imaging sequence for high blood-myocardium contrast and high imaging efficiency, the golden step’s large PE “jumps” between consecutive readouts and the induced eddy currents [42–44] compounded with the rapid intraventricular blood flow have made the technique prone to the well-known “dark flow” artifacts [45–48], which originate from bSSFP off-resonance due to the combined effects of scanner eddy current and spin flow. It was possible to avoid such artifacts by carefully adjusting the scanner field to remove or shift any off-resonance banding away from the region of interest, but it was often expected that high-flow slices would suffer from degraded image quality. This made scan planning more onerous and may restrict the use of the technique from slice orientations with more off resonance, depending on the patient anatomy.

In the current work, we introduce the sorted golden step (sGS), a significant enhancement to our original GS technique that eliminates the dark flow artifacts. Near-center readouts or pseudo-projections are still used to extract motion for self-gating, but the golden-step readouts are grouped into temporal segments and sorted according to PE before execution. This effectively reduces the PE jumps and prevents the occurrences of such artifacts while preserving the ability to track both cardiac motion and respiratory motion directly from imaging readouts. The flexibility of retrospectively reconstructing cines of arbitrary temporal resolution is also maintained, as the length of the cardia phase can be set independently as before. We also expand the application of the technique by demonstrating its effectiveness in imaging all slices of the short-axis (SAX) stack in addition to the long-axis (LAX).

## Methods

### Sequence

Building upon our prior GS work [33], a Cartesian bSSFP sequence was used, and its gradient waveforms were designed using hardware-optimized trapezoids [49] to maximize acquisition speed. The grid of PEs was indexed by *i*_*PE*_ where 1 ≤ *i*_*PE*_ ≤ *N*_*PE*_, with *i*_*PE*_ = 1 representing the most negative PE, and was to be continuously and repeatedly acquired during imaging. To start, *i*_*PE*_ = 1 was first executed as the first PE. Then, *i*_*PE*_ was incremented by *F*_*N*_, the largest Fibonacci number less than *N*_*PE*_. Note that *N*_*PE*_ can be any integer up to and including the next larger Fibonacci number, *F*_*N* + 1_. After the increment, if *i*_*PE*_ > *F*_*N* + 1_, it was wrapped with *i*_*PE*_ = *i*_*PE*_ % *F*_*N* + 1_. If the wrapped *i*_*PE*_ is greater than *N*_*PE*_, this *i*_*PE*_ was not executed but instead incremented by *F*_*N*_ again (and then wrapped) to set the next PE. For example, for a scan with *N*_*PE*_ of 192, *i*_*PE*_ would be incremented by 144 after each readout, subject to wrapping if above 233. Should the *i*_*PE*_ fall above 192, it would be incremented again by 144 before being executed.

**Fig. 1:**
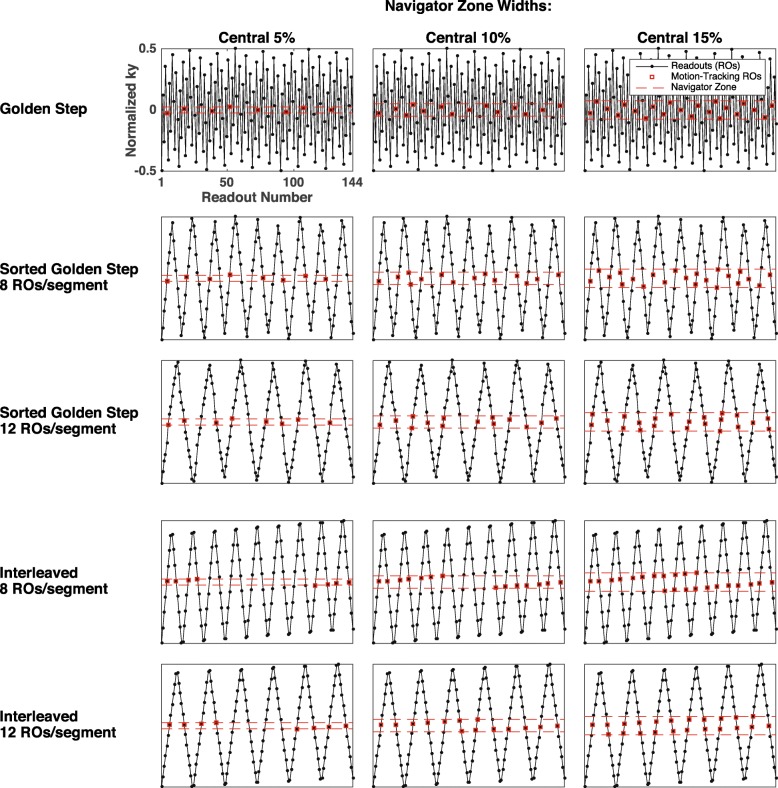
Comparison of Cartesian phase-encode (PE) schemes. In each row, a Cartesian *k*_*y*_ grid with 144 evenly spaced PEs is covered exactly once by a particular PE scheme. In each column, a central-*k*_*y*_ region of a specific width is defined as the “navigator zone” (red dashed). Low-PE readouts falling within it are considered “pseudo-projections” (red squares) and used for motion tracking. Row 1: the original integer golden-step scheme provides pseudorandom navigator-zone coverage that is approximately uniform in both time and *k*_*y*_. Row 2 and 3: the golden-step PEs are sorted into temporal segments of 8 and 12, respectively, significantly reducing PE jumps while preserving the pseudorandom coverage of the navigator zone in both time and *k*_*y*_. Row 4 and 5: for comparison, if the “interleaved” PE scheme, a commonly used segmented scheme, were to be used instead of the golden step, there would be large temporal gaps in the navigator zone coverage, i.e., there would be no PE falling inside the central-*k*_*y*_ zone (red) to sample the motion for extended periods of time (e.g. row 4 columns 1 and 2, and row 5 column 1). Those that do fall inside would also have be temporally structured (slowly drifting from negative *k*_*y*_ to positive in all scenarios in rows 4 and 5), causing slow time-varying bias in motion measurements

Under such arrangement, a grid of size *N*_*PE*_ would be fully covered once with exactly *N*_*PE*_ executed readouts. The PEs that fell within a small central portion of *k*_*y*_ (the “navigator zone”) were deemed “pseudo-projections” and would later be used for motion tracking. As shown in our previous work, incrementing and wrapping by Fibonacci numbers would ensure a pseudorandom PE coverage that was approximately uniform in both time and *k*_*y*_, inside and outside of the navigator zone. An example of the original GS can be seen in Fig. 1 (row 1).

For the sGS in this work, however, the stream of PEs were divided into temporal segments of equal size (e.g. 12 per segment) and sorted within each segment according to *k*_*y*_. Ascending sort and descending sort alternated to avoid the sudden *k*_*y*_ change between two segments. The sGS scheme is illustrated in Fig. 1, where it is also compared with the original GS and the interleaved PE scheme, a commonly used segmented Cartesian scheme. Note that the sGS may resemble the alternately sorted “up-and-down” interleaved scheme shown; however the interleaved PE scheme is prone to having large temporal gaps in the navigator zone coverage, i.e., no PEs would fall inside the central portion of *k*_*y*_ for long periods of time, during which motion could not be measured (see Fig. 1 rows 4 and 5). PEs that do fall inside the navigator zone would also have be temporally structured (e.g. slowly drifting from negative *k*_*y*_ to positive in Fig. 1 rows 4 and 5), causing slow time-varying bias in motion measurements. More extensive and systematic examinations of the properties of the sGS scheme relative to the GS and the interleaved can be found in Additional file 1, where their performance characteristics as related to both motion sampling and image formation are defined and simulated under various configurations and motion scenarios.

**Fig. 2:**
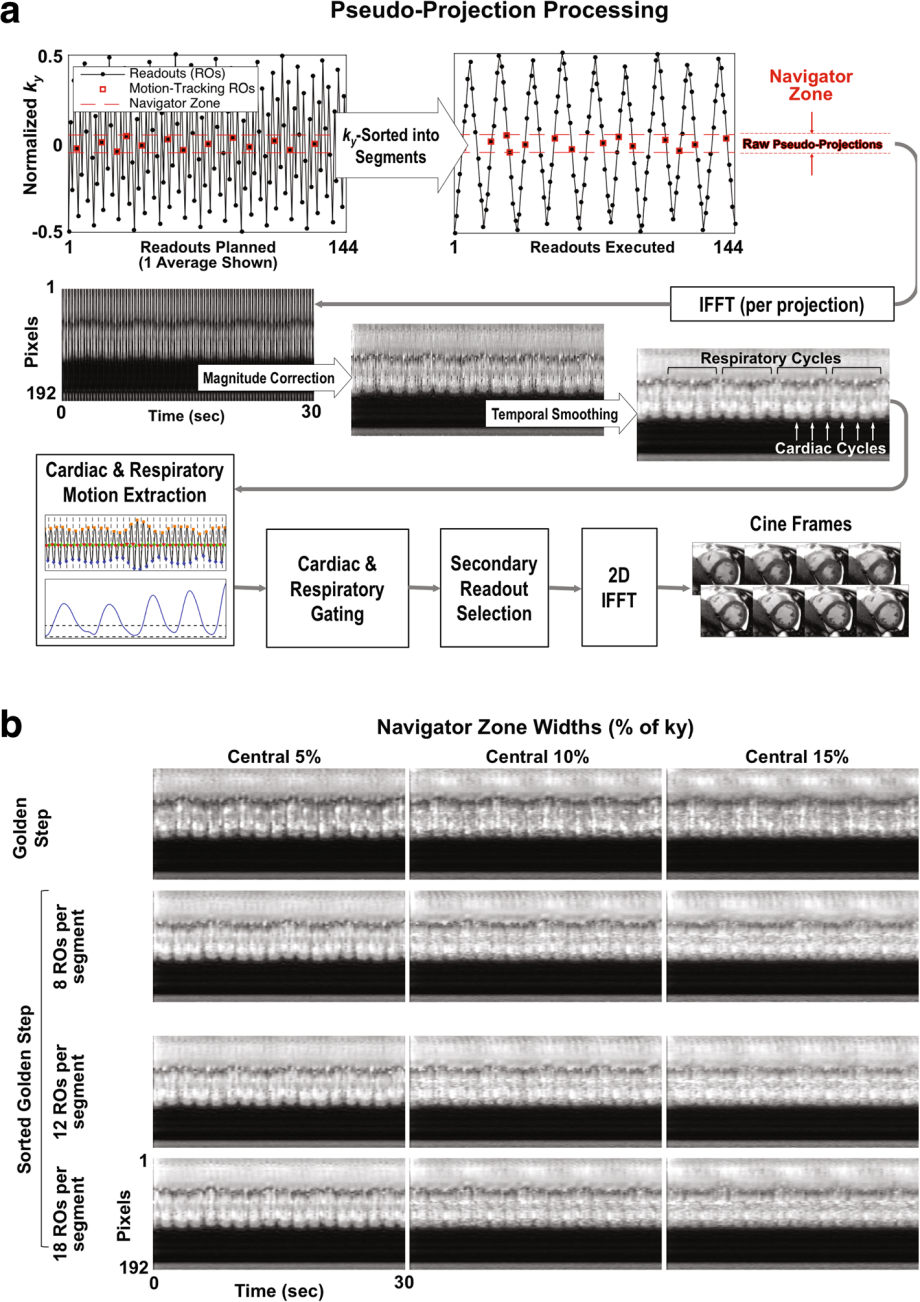
Streams of sorted pseudo-projections for motion tracking and effect of sorting-segment size. **a** The golden-step (GS) PEs are sorted by *k*_*y*_ into segments (size of 8 shown) before execution. The sorting does not affect the original GS motion tracking technique, which follows: Cartesian readouts falling within a near-zero *k*_*y*_ zone (known as the “navigator zone”) are considered “pseudo-projections.” To reveal motion, the pseudo-projections’ *k*_*y*_-dependent magnitude variation is corrected, and any remaining variation in time is smoothed. Cardiac motion and respiratory motion are extracted to guide the two-stage data selection, after which cine frames were reconstructed through simple inverse Fourier transform. **b** The effects of segment size and navigator zone width on pseudo-projections: sorted GS with various segment size are compared to the original GS at typical navigator zone widths. Both cardiac and respiratory cycles are visible at all practical segment sizes, but generally more clearly at lower navigator zone widths. Note that the streams shown here were processed to prioritize cardiac motion; respiratory motion could also be highlighted using the same data (see Figure 3). ROs: readouts

It is noteworthy that the size of the sorting segment in sGS was unrelated to the size of each cardiac phase. In other words, the flexibility of retrospectively reconstructing cines of arbitrary temporal resolution was preserved, and the motion-gated cine reconstruction process described in our previous work was unchanged (Fig. 2a).

### Imaging experiments

Five healthy subjects 29 ± 7 years; (3 females) were imaged under local institutional review board approval and with written informed consent. A 1.5 T clinical system (Avanto, Siemens Healthineers, Erlangen, Germany) was used with its standard chest and spine coils. All scans used a 35° flip angle and the standard half-flip angle half-TR bSSFP opener [50], followed by 100 dummy TRs to transition to the steady state. All imaging slices were set to a thickness of 8 mm and a readout field of view (FOV) of 300 mm covered using 192 points. The phase-encode FOV was adjusted to fit the subject size and ranged from 225 to 300 mm, covered using 144 to 192 PEs to maintain a 1.56 × 1.56 mm^2^ in-plane resolution. Depending on the slice orientation and the optimized gradient design, the TR mean ± sd was 2.78 ± 0.067 ms.

For each subject, a SAX stack of 9 or 10 slices were acquired to cover the extent of the left ventricle (LV) along with a 4-chamber LAX slice. For each slice, a segmented prospectively ECG-gated cine was acquired under breath hold as the image-quality reference (ECG BH), using a segment size of 12 readouts, resulting in a temporal resolution of approximately 33 ms per cardiac phase. Depending on the subject heart rate, 20 to 24 cardiac phases were acquired. Following the ECG BH scan, a 60-s sGS scan was acquired under free breathing (sGS FB), using a sorting segment size of 12 readouts. Finally, the original GS scan and/or sGS FB scans with other segment sizes were acquired for subjects and slices that exhibited strong dark flow artifacts so as to demonstrate through comparison how the sGS scans would effectively reduce such artifacts. All readouts were continuously acquired and saved for offline retrospective cine reconstruction.

### Motion extraction and cine reconstruction

The sGS FB scans were retrospectively reconstructed using similar techniques as in our prior work [33]. In brief, readouts within the central 5% of the *k*_*y*_-space were designated pseudo-projections and each was inverse Fourier-transformed into 1D image domain. The *k*_*y*_-dependent magnitude fluctuation in time was removed from the pseudo-projection stream by applying a *k*_*y*_ magnitude-calibration factor which described the expected magnitude ratio between the pseudo-projection at each non-zero *k*_*y*_ and the true projection at *k*_*y*_ = 0. (The calibration factor was built for each individual FB scan by averaging the magnitude of all readouts available at every *k*_*y*_ during that scan. More details in [33].) To reveal cardiac motion, the stream was further smoothed in time using a Gaussian window of a temporal full width half max (FWHM) equivalent to the acquisition time of one full k-space. A group of 10 projection pixels showing the strongest cardiac frequencies were then detected, and their group-average intensity over time was used as the cardiac gating waveform to replace the ECG. To reveal respiratory motion, the temporal smoothing was instead performed using a running averaging window of a duration equivalent to acquiring the k-space twice. Principal component analysis (PCA) was then used to identify the primary component of intensity change in time, which served as the respiratory waveform. Motion extraction of both cardiac and respiratory motion was performed over all receiver coils at once so that the most motion-indicative pixels and principal components could be selected from of all available coil data.

Cardiac events were derived from the cardiac gating waveform using a moving-average-crossing (MAC) algorithm [51]. The events corresponding to the cyclical minima of the waveform (“troughs”) were used to define the cardiac cycles due to their low timing error [33]. Each cycle was divided into the same number of cardiac phases as there were in the subject’s ECG BH scan. An acceptance window for the respiratory waveform, centered on the most-frequent respiratory position of the scan, was automatically found to accept 30% of all readouts. These readouts were binned into the cardiac phases, and then further binned by PE positions. If a cardiac-PE bin had no readout available, readouts from the two circularly adjacent cardiac phases with the same PE were copied to fill the deficiency. Then, a second layer of similarity-based data sorting was used to further select readouts: if a cardiac-PE bin contained more than one readout, only the readout whose corresponding pseudo-projection was the most similar to the mean pseudo-projection of the cardiac phase was admitted for final image reconstruction. As such, the averaging of multiple readouts within a bin was avoided for appropriate comparisons against the ECG BH images, though in practice such averaging is certainly feasible for improved signal-to-noise ratio (SNR). The filled k-space for each cardiac phase was then inverse Fourier-transformed to image domain. The imaging data from all available receiver coils were combined on a root-sum-square basis. No measure such as parallel imaging or iterative image reconstruction was used to compensate for possible missing k-space readouts, so that the motion-tracking ability of the proposed technique could be evaluated directly. For consistency, the ECG BH cines were also reconstructed offline by applying direct inverse Fourier transform to the raw k-space data of each frame, as opposed to being reconstructed directly on the scanner, where the manufacturer’s built-in filters might confound image quality.

### Image quality evaluation

Cardiac functional metrics as measured by the ECG BH and self-gated sGS FB cines were determined and compared: each stack of SAX cines were processed using Seg3D [52] where, for the end-diastolic and end-systolic frames, the LV blood pool and myocardium were semi-automatically delineated using the built-in Otsu’s method and intensity-based region growth in 3D. This yielded end-diastolic volume (EDV), end-systolic volume (ESV), ejection fraction (EF), and end-diastolic myocardial volume (EDMV).

Image sharpness of the LV blood-myocardium border was also measured for the ECG BH and sGS FB images, using an intensity profile-based method [18]: on the end-diastolic and end-systolic frames, three linear segments were drawn across the blood-myocardium border, perpendicular to the boundary, on the septal wall where it was free of papillary muscles and trabeculae carnae. On each profile, the minimal and maximal intensities were found, along with the distance (in mm) required for the intensity to transition from 20 to 80% of the spread between the minimum and maximum. The inverse of the average of the three transition distances was used as the sharpness of the image. This process was repeated for mid-ventricular SAX slices and all LAX slices.

For both functional and sharpness metrics, the paired Student’s *t*-test was used to determine any statistical difference between ECG BH and sGS FB cines, using a two-sided statistical significance threshold of 0.05. Because the *t*-test assumes data samples to be normally distributed, each data sample was standardized and compared to a standard normal distribution using the two-sided Kolmogorov-Smirnov at a significance threshold of 0.05 to determine if the usage of Student’s *t*-test was appropriate.

## Results

### Simulations

As reported in detail in the Additional file 1, the simulation results show that the sGS almost always performs as well as the GS in motion sampling, with the sGS and GS both outperforming the interleaved scheme by large margins in terms of the unbiasedness and uniformity of motion sampling. In image formation, the sGS appears to outperform both the GS and interleaved schemes in terms of k-space sampling artifact minimization.

### Motion tracking

Typical sGS pseudo-projection streams can be seen in Fig. 2, which also compares them with those generated by the original GS. Both cardiac motion and respiratory motion are visible at all sorting segment sizes. At a particular navigator zone width, different segment sizes appear to reveal motion equally well. At any segment size, a lower navigator zone width appears to better reveal motion. At 5% navigator zone width, in particular, all practical segment sizes have clearly revealed motion.

**Fig. 3:**
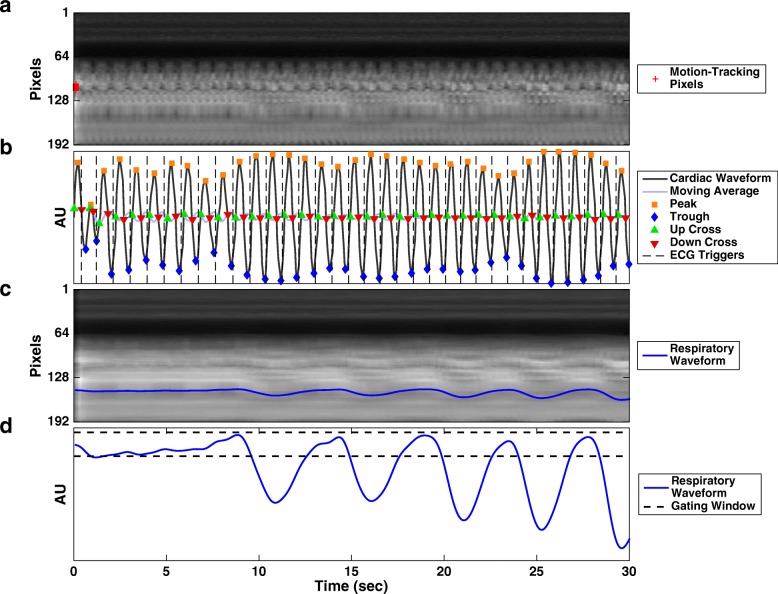
Cardiac and respiratory motion extraction from pseudo-projections. The central 5% phase encodes (pseudo-projections) were processed for optimal cardiac motion detection (**a**). From a group of automatically detected “cardiac pixels” (red “+” markers), the cardiac waveform was derived and was used to generate cardiac events (**b**) to replace ECG triggers (dashed, shown for reference). The same pseudo-projections were processed for optimal respiratory motion detection (**c**), from which a respiratory waveform was extracted using principal component analysis (PCA) to perform respiratory gating (**d**). In this experiment, the subject was instructed to breath-hold for several seconds before breathing freely. The plateau region at the beginning of (d) shows that the PCA can capture non-cyclical motion. AU: arbitrary unit

The automatic extraction of cardiac motion and respiratory motion (Fig. 3) was successful for all scanned subjects at all imaging slices. As an enhancement to our prior GS work, the same pseudo-projections were processed in two different ways to best reveal cardiac motion (Fig. 3a) and respiratory motion separately (Fig. 3c). As a result, the two types of motion were the most dominant intensity variation on their respective pseudo-projection streams and were effectively captured by the MAC algorithm (Fig. 3b) and PCA (Fig. 3d) with minimal cross-contamination.

### Image quality

**Fig. 4:**
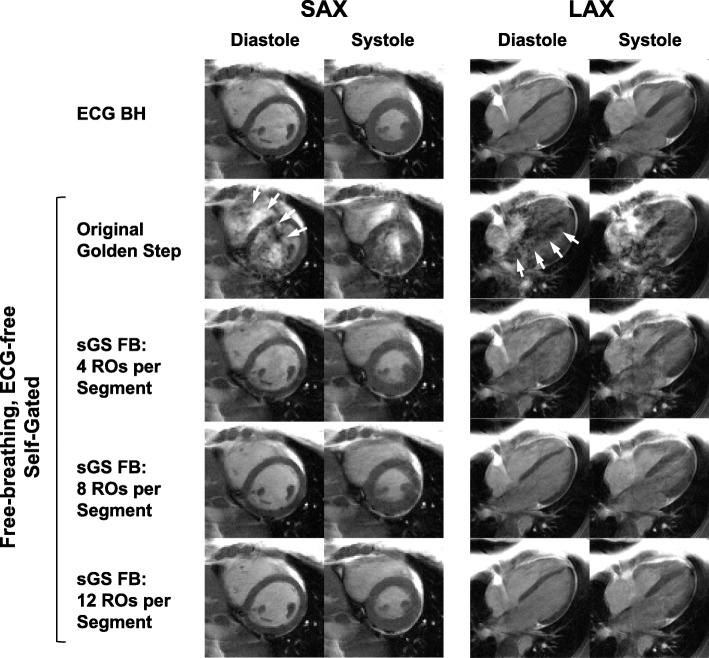
Comparison of images acquired at various number of readouts per segment. A balanced steady state free precession (bSSFP) off-resonance band near the imaging slice, which is not observed on the ECG-gated breath-hold reference cines (ECG BH, Row 1) due to its sequential PE, caused severe dark flow artifact (white arrows) with the original GS (Row 2) due to the compounding effects of large PE jumps and flow. The artifact is significantly reduced when readouts are sorted into segments of ascending or descending PEs (sGS FB, Row 3–5). At more than 4 readouts per segment, the artifact becomes essentially unnoticeable. bSSFP: balanced steady state free precession. PE: phase encode. RO: readout. SAX: short axis. LAX: long axis

**Fig. 5:**
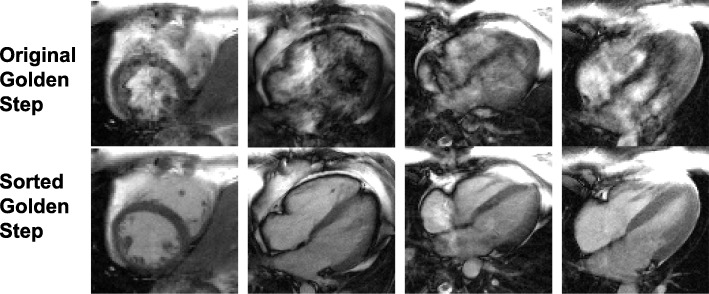
Comparison of the golden step and sorted golden step acquisitions in artifact-prone slices. In some slices of some subjects, the main field inhomogeneity and blood flow are significant enough to compound with the eddy current induced by the original golden step’s large phase-encode jumps, forming the dark flow artifact of bSSFP (Row 1). However, the same slice can be imaged virtually free of the artifact using the sorted GS (Row 2, using 12 readouts/segment). The sorted golden step (sGS) has dramatically reduced, if not entirely eliminated, the artifact in all problematic slices encountered in this study. Four such slices from three subjects are shown here

**Fig. 6:**
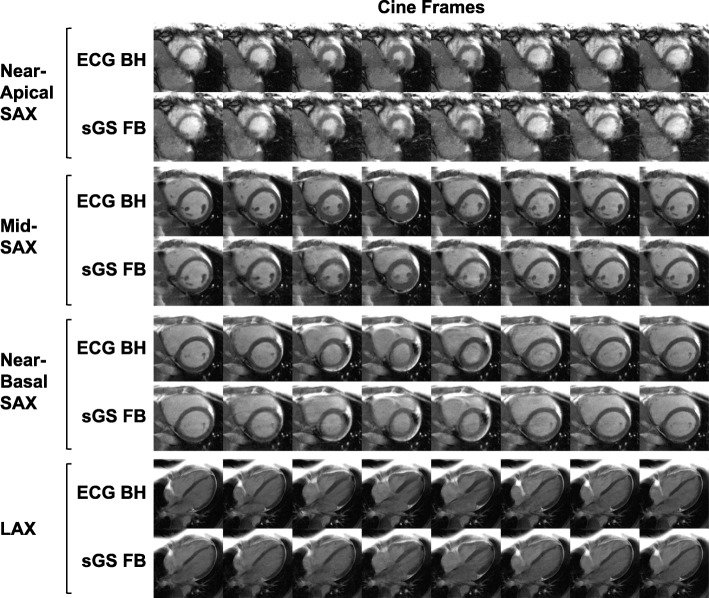
Visual comparison of cine image quality. Eight frames from a 24-frames free-breathing ECG-free self-gated sorted golden-step cine (sGS FB) acquired with 12 readouts per segment are compared with the references cine (ECG BH) acquired with ECG gating, breath hold, and sequential PEs. Several representative slices of the SAX stack at different levels (apical, mid-ventricular, basal) and a 4-chamber LAX slice are shown. Mild ghosting and blurring may be visible on some sGS images but all are free from flow- and eddy current-induced dark flow artifacts. SAX: short axis. LAX: long axis

The retrospective self-gated cine reconstruction was successful for all scanned subjects, and the reduction of the dark flow artifacts was dramatic. Even for cases with prominent artifacts (Fig. 4), sorting readouts into segments of 4 immediately eliminated most of the artifacts. At 8 or more readouts per segment, the artifacts were unnoticeable. The artifact reduction of several additional instances intentionally chosen for severe artifacts are shown in Fig. 5. The same reduction held for all cardiac phases and both SAX and LAX slices (Fig. 6), resulting in high-quality sGS FB images as compared to the ECG BH scans. The clear visualization of small features such as the trabeculae carnae demonstrates the effectiveness of motion tracking.

**Fig. 7:**
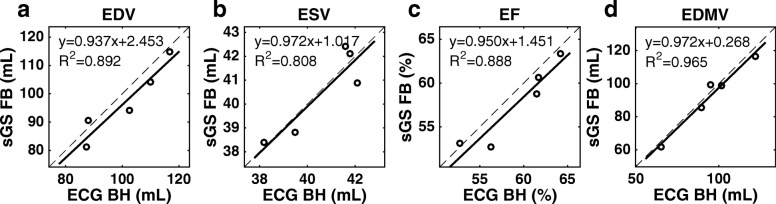
Comparison of LV functional metrics as measured from ECG BH and sGS FB images. As measured from the SAX stacks, the end-diastolic volume (EDV, **a**), end-systolic volume (ESV, **b**), ejection fraction (EF, **c**), and end-diastolic myocardial volume (EDMV, **d**) of the free-breathing self-gated sorted golden-step (sGS FB) images show good agreement with those of the ECG-gated breath-hold references (ECG BH), with average absolute errors of approximately 5% or less. LV: left ventricle

**Fig. 8:**
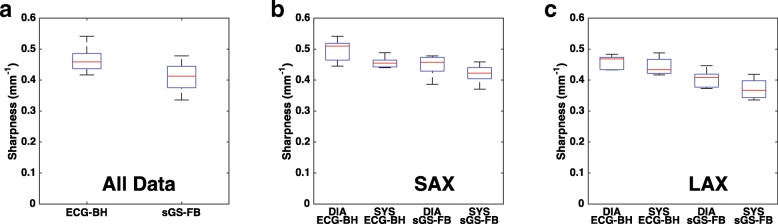
Comparison of LV blood-myocardium sharpness as measured from ECG BH and sGS FB images. Whether combined (**a**) or separated into SAX (**b**) and LAX (**c**) groups, the sGS FB images were in general slightly less sharp than the ECG BH scan, although the differences are not large. SAX: short axis. LAX: long axis. DIA: end-diastole. SYS: end-systole. LV: left ventricle

Quantitatively, the cardiac functional metrics derived from the sGS FB scans showed good agreement with those from the ECG BH scans (Fig. 7). The two kinds were found to differ by less than 1 ml on average for SAX end-systolic images, and less than 4 ml on average overall. Such difference was found not to be statistically significant (at a threshold level of *P* = 0.05) by the paired *t*-test (Table 1), although some *P* values were marginal. The blood-myocardium sharpness measures of the sGS FB images were in general slightly reduced relative to those of the ECG BH scan (Fig. 8, Table 2), but the average difference was less than 0.04 mm^− 1^ for SAX end-systolic images and less than 0.075 mm^− 1^ overall. The end-systolic sharpness in general was less than that of end-diastole, presumably due to the former’s high rate of myocardial motion. As shown in Table 2, the difference in sharpness was found to be statistically significant. Each data sample was tested using the Kolmogorov-Smirnov test and found not to be significantly different from being normally distributed.

**Table 1:** LV functional metrics measured from ECG BH and sGS FB images

	EDV (ml)	ESV (ml)	EF (%)	EDMV (ml)
ECG BH	100.9 ± 13.0	40.6 ± 1.7	59.3 ± 4.6	94.7 ± 20.6
sGS FB	97.0 ± 12.9	40.5 ± 1.8	57.7 ± 4.7	92.3 ± 20.4
Difference	3.9 ± 4.3	0.1 ± 0.8	1.5 ± 1.6	2.4 ± 3.9
*P* value (*t*-test)	0.111	0.757	0.097	0.238

*ECG BH* ECG-gated breath-hold reference cines, *sGS FB* free-breathing self-gated sorted golden step cines, *EDV* end-diastolic volume, *ESV* end-systolic volume, *EF* ejection fraction, *EDMV* end-diastolic myocardial volume, *LV* left ventricle

**Table 2:** LV blood-myocardium sharpness of ECG BH vs. sGS FB images

Sharpness (mm^− 1^)	SAX DIA	SAX SYS	LAX DIA	LAX SYS
ECG BH	0.496 ± 0.038	0.457 ± 0.019	0.458 ± 0.023	0.444 ± 0.030
sGS FB	0.447 ± 0.037	0.420 ± 0.032	0.403 ± 0.030	0.372 ± 0.034
Difference	0.048 ± 0.023	0.036 ± 0.020	0.055 ± 0.018	0.073 ± 0.033
*P* value (*t*-test)	0.010	0.015	0.003	0.008

*ECG BH* ECG-gated breath-hold reference cines, *sGS FB* free-breathing self-gated sorted golden step cines, *SAX* short axis, *LAX* long axis, *DIA* end-diastole, *SYS* end-systole, *LV* left ventricle

## Discussion

We have introduced a significant improvement to the original GS that is free of the dark flow artifacts and capable of producing high-quality self-gated cines. As with the original golden step, both cardiac motion and respiratory motion can be captured at no additional cost at acquisition time. While many other pseudo-random and golden angle-inspired PE techniques [35–41] have been reported, all were reported on radial imaging. Therefore it is important for the sGS to demonstrate its effectiveness in Cartesian imaging, while allowing both motion tracking and artifact reduction, no less.

A key advantage of the original GS is minimal motion-related pre-scan planning: the navigator zone can be retrospectively set to arbitrary widths and it is always pseudo-randomly and evenly covered by readouts. This feature is preserved in the proposed technique, except for a single parameter: the number of readouts per sorting segment needs to be determined prior to the scan.^1^ However, in our experience, 8 readouts per segment would effectively eliminate even very severe dark flow artifacts (Fig. 4). Thus 8 can be used as a default setting for most scans. Should an imaging slice be found particularly problematic, i.e. a bSSFP band in or near the imaging slice is confirmed with interleaved-PE test scans [46–48] and does not respond well to shimming or frequency shifting, then 12 or 16 readouts per segment can be used instead. One can freely choose a segment size for each scan in an imaging study, knowing all scans can be reconstructed using the same motion-extraction pipeline. Given how frequently banding and dark flow artifacts appear in cardiac imaging and how they would restrict the use of the original GS, the requirement to choose the segment size prior to scanning is a small compromise.

### Limitations

The primary limitation of this work arises from the fact that the effectiveness of sGS was demonstrated on a relatively small pool of five healthy subjects which represents is a very limited dataset. Though the complete SAX stack and a 4 chamber LAX view were tested, these measurements are not statistically independent values, greatly limiting the effective number of samples. Furthermore, no patient data are presented as part of this validation of the sGS method. Though it is important to note that sGS is an extension of the previously published method, and that the method worked well in all SAX and LAX views, more data spanning a broader range of pathological flow is needed to verify sGS is effective in both motion tracking and artifact reduction.

As with our previous GS work, only the 4-chamber view was scanned for LAX slices primarily because it generally captures the greatest amount of in-plane blood flow among all LAX slices and therefore was by far the most challenging artifact-wise in our experience. Because other LAX views are not expected to have more in-plane flow, the 4-chamber view represents a reasonable demonstration of the artifact-reduction efficacy of sGS. Motion-tracking wise, all LAX slices have adequate in-slice blood-volume change (much more than the apical-SAX slices, which were included in this work) to reveal cardiac motion on the pseudo-projections. Also, all LAX slices are by definition similarly aligned with the superior-inferior direction, and thus will be able to reveal respiratory motion as does the 4-chamber view. In other words, the sGS motion tracking would in all likelihood perform equally well for other LAX slices as reported in this work.

Prospectively ECG-gated scans were used as the reference cines in this work, whereas in clinical practice, retrospective ECG gating is the standard, because patients are often arrhythmic with variable RR intervals. But since healthy subjects were used in this work, capturing the end-diastolic period was not difficult and only a small portion of the cardiac cycle might have been missed. We would not expect any changes in measured volumes because of the use of prospective ECG gating.

During post-processing, the separate generation of cardiac and respiratory pseudo-projection streams (Fig. 3 a and c) reduced cross-contamination in the automatically extracted motion waveforms. For the temporal smoothing of pseudo-projections, a simple Gaussian window and a simple running-average window were used for cardiac motion and respiratory motion, respectively. Although one can design digital filters that adapt to the subject’s heart rate and respiratory rate, in our experience the simple fixed windows were quite adequate in performance. In clinical settings, subject-dependent settings and parameters are time-consuming and error-prone, therefore if a one-size-fits-all solution works well, it is strongly preferred and is in fact an indication of robustness of sorted pseudo-projections. It is true that, should this technique be used for applications beyond cardiac and respiratory motion tracking, more adaptive filtering may be needed. For the scope of this work, however, the effectiveness of simple fixed smoothing filters supports the strength of sorted pseudo-projections for cardiac-respiratory dual self-gating, which is among the most challenging motion tracking objectives in MRI.

Similarly, the simpler principal component analysis was used to extract cardiac motion and respiratory motion, whereas CMR literature has reported more sophisticated and effective motion-detection techniques such as independent component analysis (ICA) [53–55]. As with our previous GS work, we have chosen a simple and straightforward motion separation technique to show that the sGS is capable of providing sufficient motion-tracking information so that the entire reconstruction process would work end-to-end, even with this relatively primitive technique to derive motion.

During k-space data selection for image reconstruction, only one readout was admitted by similarity-based data sorting to each PE position of each cardiac phase. This was intended to maintain a fair comparison with the ECG BH images, which only had only one readout per PE position. In practice, if the scan length provides abundant data relative to the number of cardiac phases reconstructed, more than one readout can be admitted, at least to some of the PE positions. By averaging multiple readouts per PE position, the final image SNR is boosted, though this may come at a cost to image sharpness. Because respiratory motion is complex, even with gating and similarity-based data sorting, some discrepancy in motion state may be present among the multiple admitted readouts per PE. However, with the flexibility of golden step, one can arbitrarily control the scan length and data availability, either at scan time or at reconstruction time (using arbitrary subsections of the scan for reconstruction). In essence, one can freely and smoothly trade off image SNR against sharpness. In a similar way, PE positions that remain unpopulated could be filled using generalized autocalibrated partially parallel acquisitions (GRAPPA) [56] or similar k-space driven parallel imaging techniques.

As with most published free-breathing cine techniques, the image sharpness of the gated free-breathing scans was slightly lower than the breath-hold reference due to the complex nature of the respiratory motion, which ideally would require much more complex approaches to address rather than simple gating, and may well be a separate area of research on its own [20, 57–60]. However, coming at no acquisition cost, the performance of respiratory tracking in this work is robust, and the high accuracy of sGS-derived functional metrics suggests that the bulk of routine CMR exams can benefit from this technique.

### Extensions and potential

For the sGS, the length of the sorting segment has been a key (and the only) question of parameter choice. Namely, although a high number of readouts per segment would in principle lead to even smaller *k*_*y*_ discontinuities between readouts, it would also result in large temporal gap in the coverage of the navigator zone and is therefore not advisable. This choice is closely related to that of the navigator zone width, as a wider navigator zone may alleviate the large gaps to some degree. However, as Fig. 2 shows, a high navigator zone width may result in poor depiction of motion, presumably because high-*k*_*y*_ readouts contain weaker signal relative to noise, and scaling up their magnitude during magnitude correction could amplify noise. Further, as discussed in detail in our prior GS work [33], the navigator zone width affects spatial resolution of motion tracking, as readouts with different *k*_*y*_ values are sensitive to tracking objects of different sizes. Given all these tradeoffs, the present work used the same 12 readouts per sorting segment and navigator zone width of 5% for all scan subjects with successful results. While such parameter choice has been effective for cardiorespiratory motion tracking, others who wish to apply the technique to other types of motion tracking may likely need to tune the sorting-segment length and navigator zone width, according to the size of the object and the frequency of the motion. In fact, although a constant boundary for the navigator zone appears to work well in this work and our previous GS work, a dynamic navigator zone can be devised to temporarily relax or tighten the zone boundary depending on the current temporal gap in navigator zone coverage, so as to both improve temporal coverage and reduce noise amplification.

It is natural to consider extending the sorting approach of this work to the original radial golden angle [34] to reduce the angular jump between consecutive radial readouts. Indeed, alternative approach has been reported for that purpose [tiny GA], although the great majority of radial cardiac imaging works [17, 19, 41, 61–63] do not report problematic angular jump or dark-flow artifacts. Interestingly, studies specifically targeting such artifacts only address Cartesian imaging [46–48, 64, 65]. Radial imaging appears to be much less vulnerable to such artifact, presumably due to the fact that, unlike Cartesian imaging, it lacks a single phase-encode direction that constantly experiences large gradient changes. This makes it unlikely for extraneous phase to build up on spins flowing in the phase-encode direction. Any PE-jump artifact that does build up would be smeared in hundreds of radial directions instead of a single PE direction in Cartesian imaging. The artifacts would be even less noticeable given the heavy over-sampling of the center of k-space in radial imaging. Considering these reasons, radial imaging may have little need for and receive little benefit from angular sorting.

While the present study was carried out at 1.5 T field strength, we do expect that 3 T Cartesian scans involving significant flow can similarly benefit from PE sorting. Although the main field strength is not a direct contributor to the dark flow artifacts, any field inhomogeneities and off-resonance do become more pronounced on 3 T (especially considering cardiac cine scans are almost always based on steady-state imaging), further contributing to extraneous phase buildup that ultimately leads to such artifacts. PE sorting can certainly reduce any gradient-related disturbance to the spin phase, although whether the artifact reduction is even more significant than on 1.5 T requires a 3 T sGS study.

It is noteworthy that the proposed technique can be adapted to perform non-rigid respiratory motion correction. With GS, in any temporal portion of the scan, the *k*_*y*_-space is approximately uniformly populated. Without any changes to the acquisition strategy, one may run a narrow sliding window of, for example, 72 readouts (≈200 ms) across the entire scan and reconstruct a low-SNR image for each window position with generalized image-based parallel imaging (e.g. generalized sensitivity encoding [66]) using through-time summation to generate robust coil sensitivity profiles, or with compressed sensing techniques that enforce temporal sparsity. Such images may not be of diagnostic quality but can be used for non-rigid image registration. Thus, one can obtain non-rigid deformation state at any point during the scan and may use such information to correct imaging data acquired around that point. Comparing to respiratory gating, which discards a large amount of data outside the gating window, the motion correction would allow more data to be used in image reconstruction, therefore shortening total scan time and/or enhance image SNR. Moreover, cardiac self-gating can be simultaneously performed, using the same data stream and same process as described in the present work.

Overall, in the present work the sGS has been able to successfully track both cardiac and respiratory motion and yield self-gated images of comparable quality to the ECG BH cines at all attempted imaging slices. More importantly, the sGS is free of the frequently observed bSSFP dark flow artifacts, which were mild in about half of the SAX slices and rather prominent in the majority of the LAX slices in our prior experience with GS [33]. Thus, whereas previously the original GS may have been restricted to subjects and slices where the artifacts were minimal, the sGS is not restricted to any particular orientation or slice and can therefore be applied to broader clinical use.

## Conclusions

A significant improvement has been made to the Cartesian GS pseudo-projection imaging technique. The sGS technique has maintained the flexibility of GS and its ability to produce high-quality cardiac function studies from FB ECG-free scans, without the artifacts induced by the combination of eddy currents and flow originally observed in both SAX and LAX imaging orientations. This development greatly enhances the reliability and usability of the Cartesian GS in CMR imaging.

## Additional file

Additional file 1: Simulation of Sorted Golden-Step (sGS): Comparison with Golden Step (GS) and Interleaved Phase Encode Ordering. A quantitative comparison of the three acquisition orders incorporating a range of heart rates and heart rate and respiratory rate variability shows that the sGS performs almost always as well as the GS in motion tracking sampling. sGS and the GS also both outperform interleaved sampling by large margins in terms of the unbiasedness and uniformity of sampling. In image formation, the sGSoutperforms both the GS and interleaved sampling in terms of k-space sampling artifact minimization. (DOCX 564 kb)

## Abbreviations

BH: Breath hold
bSSFP: Balanced steady-state free precession
CMR: Cardiovascular magnetic resonance
ECG: Electrocardiogram
EDMV: End-diastolic myocardial volume
EDV: End-diastolic volume
EF: Ejection fraction
ESV: End-systolic volume
FB: Free breathing
FOV: Field of view
FWHM: Full width half maximum
GS: Golden step
LAX: Long axis
LV: Left ventricle/left ventricular
MAC: Moving average crossing
PCA: Principal component analysis
PE: Phase encoding
SAX: Short axis
sGS: Sorted golden step
SNR: Signal-to-noise ratio
TR: Repetition time

## Footnotes

1 It should be noted that the temporal width of each cardiac phase can still be set to arbitrarily after the scan, because it is unrelated to the number of readouts in the sorting segment here.
