# Supplementary material for: Sorted Golden-step phase encoding: an improved Golden-step imaging technique for cardiac and respiratory self-gated cine cardiovascular magnetic resonance imaging
Source: J Cardiovasc Magn Reson. 2019 Apr 18;21:23. doi: 10.1186/s12968-019-0533-8 (PMC6472023; doi:10.1186/s12968-019-0533-8)
Supplement: Supplementary file 1 — Simulation of Sorted Golden-Step (sGS): Comparison with Golden Step (GS) and Interleaved Phase Encode Ordering. A quantitative comparison of the three acquisition orders incorporating a range of heart rates and heart rate and respiratory rate variability shows that the sGS performs almost always as well as the GS in motion tracking sampling. sGS and the GS also both outperform interleaved sampling by large margins in terms of the unbiasedness and uniformity of sampling. In image formation, the sGSoutperforms both the GS and interleaved sampling in terms of k-space sampling artifact minimization. (DOCX 564 kb) [file 12968_2019_533_MOESM1_ESM.docx]

**Simulation of Sorted Golden-Step (sGS): Quantitative Comparison with Golden Step (GS) and Interleaved Phase Encode Ordering**

In addition to the illustrative example of the sorted golden step (sGS) phase-encode (PE) scheme relative to the golden step (GS) in our previous work [1] and the interleaved scheme (Figure 1 of the main text), we demonstrate here through quantitative simulations the advantages of the sGS over these two PE schemes. There are no other classes of PE schemes that we are aware of for self-gated Cartesian cine imaging. Specifically, we demonstrate through simulations, the strengths of *k*-space coverage of sGS in the context of 1) motion tracking and 2) image formation after retrospective gating of data.

## Motion Tracking

### Simulation Setup

We simulate each complete repetition of the *k*-space using the interleaved, the GS, and sGS schemes (**Figure 1A**). Because motion is measured from the pseudo-projections, i.e. normal Cartesian readouts from within the navigator zone, we focus on the navigator zone at various zone widths and examine several characteristics (below) of the PEs within that are important for motion tracking:

1. **Bias or modulation in *k_y_*:** As discussed in our original work GS [1], the raw stream of acquired pseudo-projections carries a strong *k_y_*-dependent modulation that far overpowers any measured cardiorespiratory motion, making it impractical for motion-tracking algorithms to process. Even though the modulation can largely be removed by a patient-tuned correction factor before motion tracking, any bias or modulation in PE must be minimal. (An example of such bias in *k_y_*, due to the use of the interleaved PE scheme, leading to the drifting morphology of cardiac projection is shown in **Figure 2**. The interleaved PE ordering is not pseudo-random enough for the correction factor to be tuned for the patient in practical scan durations.) During the simulation, the bias in *k_y_* is measured using:
   1. **The cumulative *k_y_*** of pseudo-projections from the beginning of acquisition to the current pseudo-projection. In order to minimize *k_y_* bias, the cumulative *k_y_* should remain close to *k_y_*=0 throughout a scan.
2. **Uniform sampling in both navigator zone (*k_y_*) and in time:** “Uniform” here is used in the sense that sampling “does not concentrated in one region” or “does not leaving large gaps” in either *k_y_* or time. This is to ensure motion is measured from various *k_y_* values and updated regularly in time, and indirectly, this also minimize the *k_y_* bias of the pseudo-projections at all time. For the simulation:
   1. **The largest remaining *k_y_* gap** in navigator zone up to the current pseudo-projection is used to measure the uniformity in *k_y_*.
   2. **The gap time** since the previous pseudo-projection sampling is used to measure the uniformity in time.

### Simulation Results

1. **Bias or modulation in *k_y_*** – The interleaved PE scheme shows an obvious *k_y_* structure that steadily drifts to the positive direction within the navigator zone at all tested zone widths (**Figure 1B**), whereas the GS and sGS do not. In practice, such drift results in the shifting morphology of motion as measured by the pseudo-projection stream (**Figure 2**). The bias of the interleaved PE is further illustrated by plotting the cumulative *k_y_* over time within the navigator zone (**Figure 1C**).
2. **Uniform sampling in both navigator zone (*k_y_*) and in time:** When uniformity in *k_y_* is shown by plotting the largest remaining gap in navigator zone *k_y_* values (**Figure 1D**), the interleaved scheme tends to neglect a certain region of the *k_y_* by leaving the largest *k_y_* gap unfilled for quite some time. The sGS and GS, on the other hand, both reduces the largest gap much faster in comparison. Temporally, the interleaved scheme tends to reduce the largest remaining *k_y_* gap at a slower pace than GS and sGS. When uniformity in time is shown by plotting the running gap time in motion sampling (**Figure 1E**), the interleaved scheme tends to leave long gaps at low navigator zone widths. The sGS and GS, again, show similarly low time gaps.

In summary, the navigator-zone simulations support our experience that the sGS offers low-bias motion sampling in both *k_y_* and in time at least as well as the GS scheme, and obviously better than the interleaved scheme.

## Image Formation

For image formation, we create another set of simulations for the entire *k_y_*-space, with the focus of measuring the data-filling completeness of *k*-space for all cardiac phases after retrospective motion-gated data rejection. Data-filling completeness is arguably the top factor impacting the quality of self-gated imaging, due to its high demand on the speed of data acquisition. Although motion blurring and temporal resolution can be considered other top factors, in self-gated imaging however, they are practically one and the same as data filling: a tighter gating window would enhance temporal resolution and eliminate motion blurring, but it would also exacerbate the challenge of data filling.

Given the wide variations in patient motion and patient habitus, it is very likely that some *k*-space views will remain unfilled given the limited scan time of a self-gated scan. The unfilled *k_y_* gaps create a superimposed binary pattern over the *k_y_* grid, corrupting the resultant image by convolving it with the inverse Fourier transform of the incomplete *k_y_* grid, known as the point-spread function (PSF). (Note that, when the *k_y_* grid is complete, its resultant PSF in image space is a delta function, which has no effect when convolved with the image.) As a result, certain formations of missing *k_y_* views (e.g. those that form a contiguous block in *k_y_* or those that appear in regular *k_y_* intervals) are particularly detrimental to image quality (because they translate to, e.g., sharp sinc-shaped or comb-shaped modulation, respectively, along *y* in image domain). Therefore, a PE scheme would preferably be able to minimize the corruption of image quality despite some missing views.

The theories of such sampling-induced corruption have been discussed in great details (e.g. [2–5]) and are beyond the scope of this writing. However, we are able to show through simulations the advantage of the sGS over both GS and interleaved in terms of sampling artifact reduction, using a few fundamental metrics that quantify the degree of image corruption. Specifically, we measure the *y*-PSF, which is evaluated in terms of its deviation from the ideal form (the delta function). We show through simulation that such advantage is not random but consistent under virtually all motion and gating conditions. More details are described below.

### Simulation Setup

Cardiac- and respiratory-gated free-breathing scans are simulated repeatedly, each time with a randomly generated motion pattern, and data acquisitions driven by the sGS, GS, and interleaved PE schemes are applied. To compare their performance, a few performance metrics are calculated throughout the scan. For each simulated scan:

- Cardiac cycles are generated using a varying heart rate, where each RR interval is a normally distributed random variable with a mean randomly drawn (uniformly) from 600 to 1000 ms (equiv. to 60 to 100 beats per minute), and a standard deviation equal to 10% of the mean. Each cardiac cycle is divided into 24 uniformly spaced cardiac phases.
- Respiratory cycles are generated using a varying rate, where each cycle is also a normal random variable with a mean randomly drawn (uniformly) from 15 to 24 cycles per minute, and a standard deviation equal to 10% of the mean. The first 20% of each respiratory cycle is accepted. (With the random shift described below, this simulates a respiratory gating window accepting a randomly placed 20% portion of each respiratory cycle, regardless of the actual shape of the respiratory waveform.)
- A random relative phase shift between the cardiac and respiratory waveforms is added by time-shifting the cardiac train by a normal random variable with mean of 0 and standard deviation equal to the mean duration of all cardiac cycles.
- Data accepted by gating is distributed to the cardiac phases, and to the appropriate positions on the 144-PE *k_y_* grid for each phase, according to sGS, GS, or interleaved PE scheme.
- Two *k_y_* completeness metrics are computed continuously throughout each scan:
  - **PSF Leak** – the sum of intensity (arbitrary unit) along *y* outside *y* = 0:
    - Ideally zero, lower value means higher image quality
    - Mean and max across all cardiac phases are both reported.
  - **PSF SNR** – the ratio (in dB) of intensity at *y* = 0 to total intensity outside *y* = 0:
    - Ideally infinity, higher value means higher image quality.
    - Mean and max across all cardiac phases are both reported.
- Total area under curve (AUC) for the duration of the scan is found for each metric.
- The simulated scan time is **intentionally** kept short (1 min) for each scan, because if the scan time is sufficiently long, any PE scheme can almost certainly fill the *k_y_*-space completely (given some random variation in cardiorespiratory rhythms).

One hundred such scans are simulated at various random cardiac and respiratory rates, and AUC of the metrics are plotted on scatter plots, where the *x*- and *y*-axes are the mean cardiac and respiratory rates for each scan.

### Simulation Results

A typical example of one simulated scan is shown in **Figure 3**. Cardiac cycles and respiratory cycles with the random jittering and random phase shift (**Figure 3A**) interact with all three PE schemes, and the resultant k-space completeness metrics over the first minute are reported in **Figure 3B – E**. In this example, for the PSF leak (lower is better), both the cardiac-phase mean (**Figure 3B**) and cardiac-phase max (**Figure 3C**) are the lowest for sGS throughout the scan. For PSF SNR (higher is better), both cardiac mean (**Figure 3D**) and max (**Figure 3E**) are the highest among the three throughout the scan.

In fact, in all 100 scans simulated with various cardiorespiratory rates and phase shifts, the PSF leak metrics consistently remain the lowest of the three schemes (**Figure 4A**, **B**) while the PSF SNR metrics consistently remain the highest of the three (**Figure 4C**, **D**)

In summary, in all simulated scans, the sGS show the best k-space completeness metrics among all three PE ordering schemes. This lends strong support of the sGS’s optimality in image formation when it comes to cardiac- and respiratory self-gated cine scans.

## Conclusion

The simulation results show that the sGS performs almost always as well as the GS in motion tracking sampling. The sGS and the GS also both outperform the interleaved scheme by large margins in terms of the unbiasedness and uniformity of sampling. In image formation, the sGS appears to outperform both the GS and interleaved schemes in terms of k-space sampling artifact minimization.


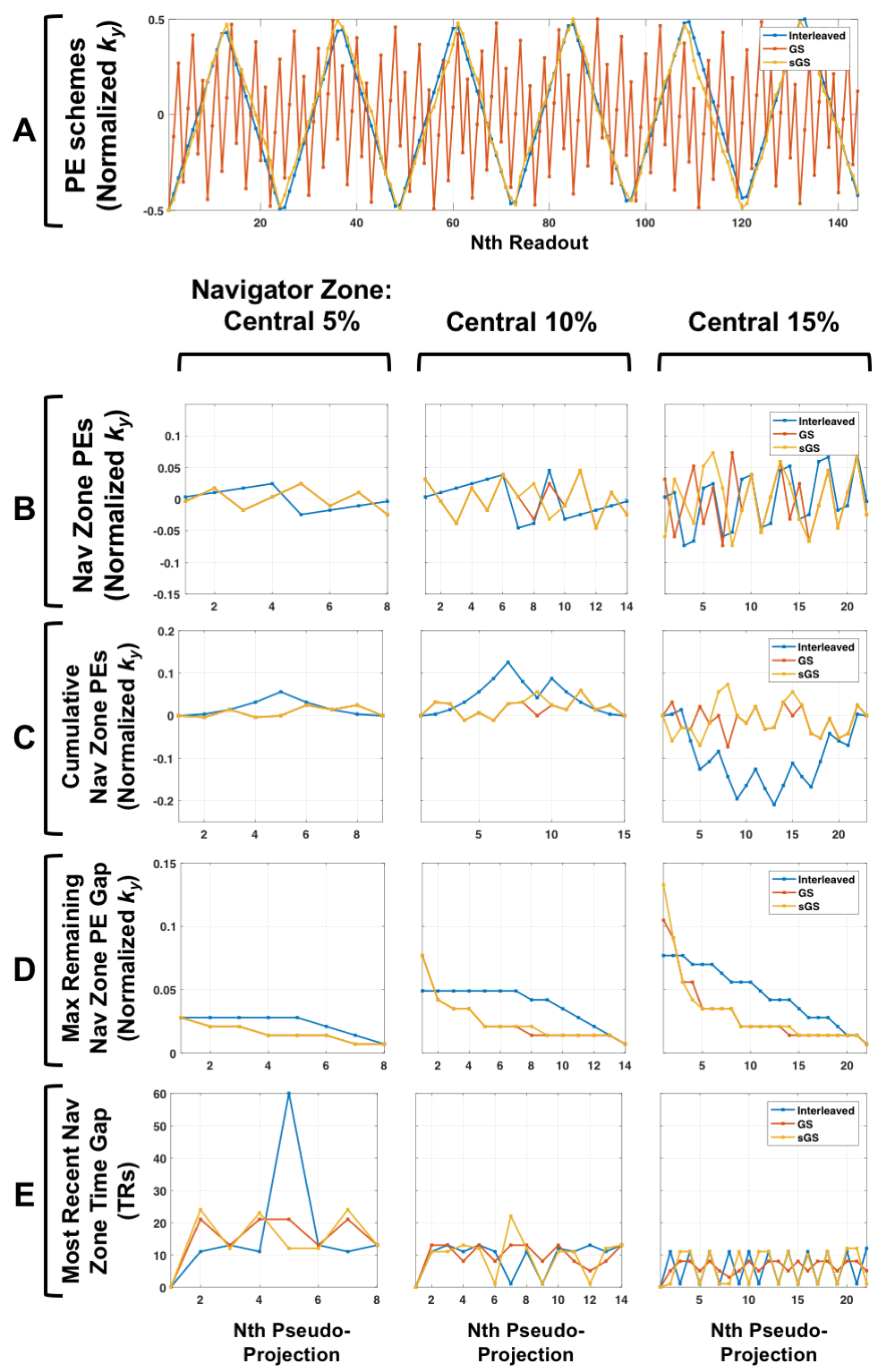


Figure 1. Comparison of the navigator-zone characteristics of the interleaved, golden step (GS), and sorted golden step (sGS) PE schemes. Row A: the three schemes over one complete acquisition of a 144-PE *k_y_* space. Row B: PEs inside the navigator zone. Row C: cumulative *k_y_* of navigator-zone PEs, note that both GS and sGS remain close to *k_y_*=0, indicating the symmetric and uniform coverage of the *k_y_* interval of the navigator zone. In comparison, the interleaved scheme deviates far from 0 at higher navigator zone widths, suggesting a bias in the sampling of *k_y_*, which leads to time-varying depiction of motion. Row D: the largest remaining *k_y_* gap within the navigator zone over time, note that just like GS, the sGS quickly reduces the gap size, much faster than the interleaved. Row E: time intervals between two measurements of the navigator zone, note that the GS and sGS do not have large intervals as does the interleaved, and generally have smaller fluctuation than does the interleaved scheme.


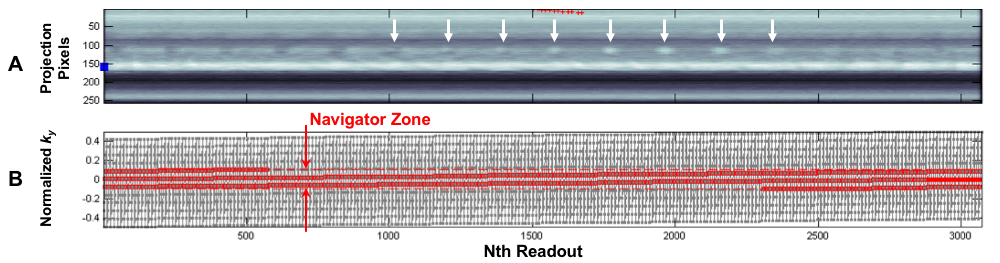


Figure 2. An example of the inconsistent morphology of cardiac motion: (A) The changing appearance of cardiac cycles (white arrows) on the magnitude-corrected pseudo-projections (red squares) during the course of a short breath-hold scan (approx. 10 sec). (B) The interleaved PE ordering used to acquire the scan exhibits a structural bias by having a slow drift from negative to positive *k_y_* within much of the navigator zone (red dashed).


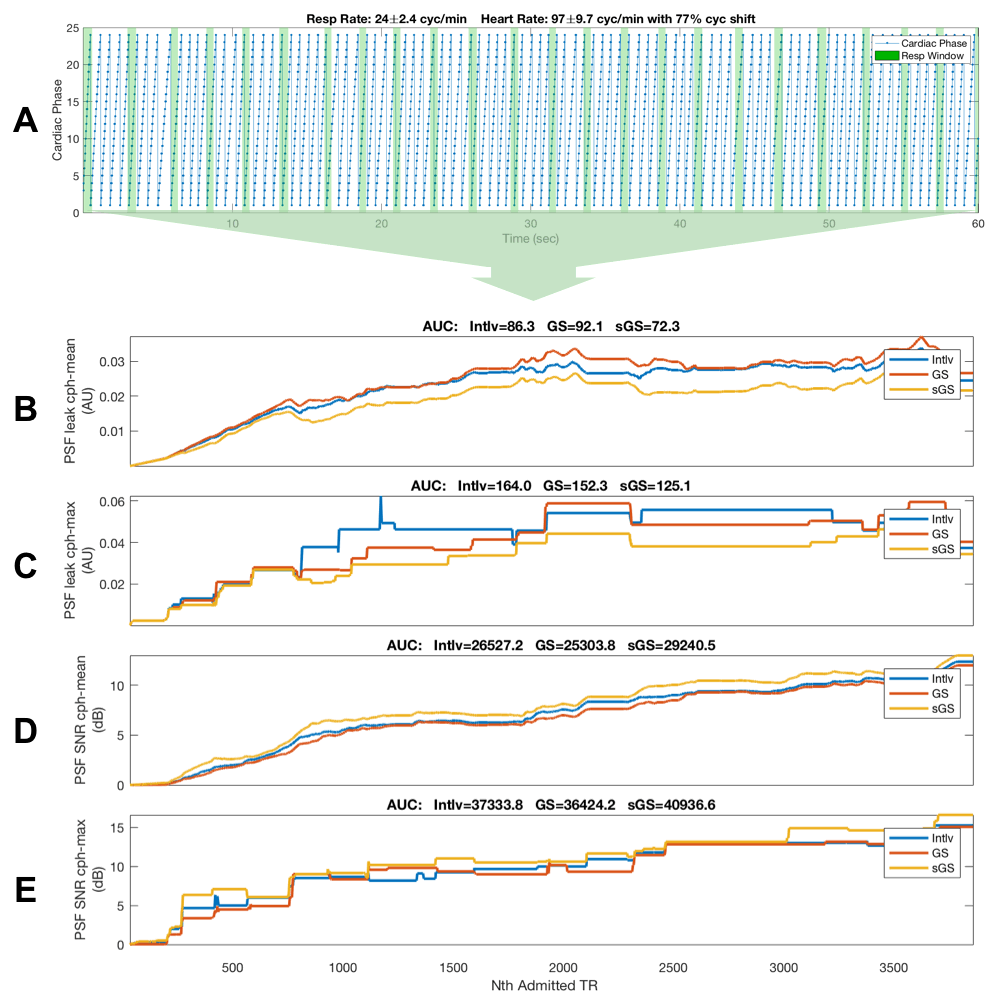


Figure 3. A typical example of a simulated cardiorespiratory self-gated scan: Cardiac cycles and respiratory cycles with random jittering and random phase shift (A) interact with all three PE ordering schemes. During the accepted respiratory windows (green shading), the resultant k-space completeness metrics over time are plotted: PSF leak (lower is better) mean (B) and max (C) across all cardiac phases, and PSF SNR (higher is better) mean (D) and max (E) across all cardiac phases.


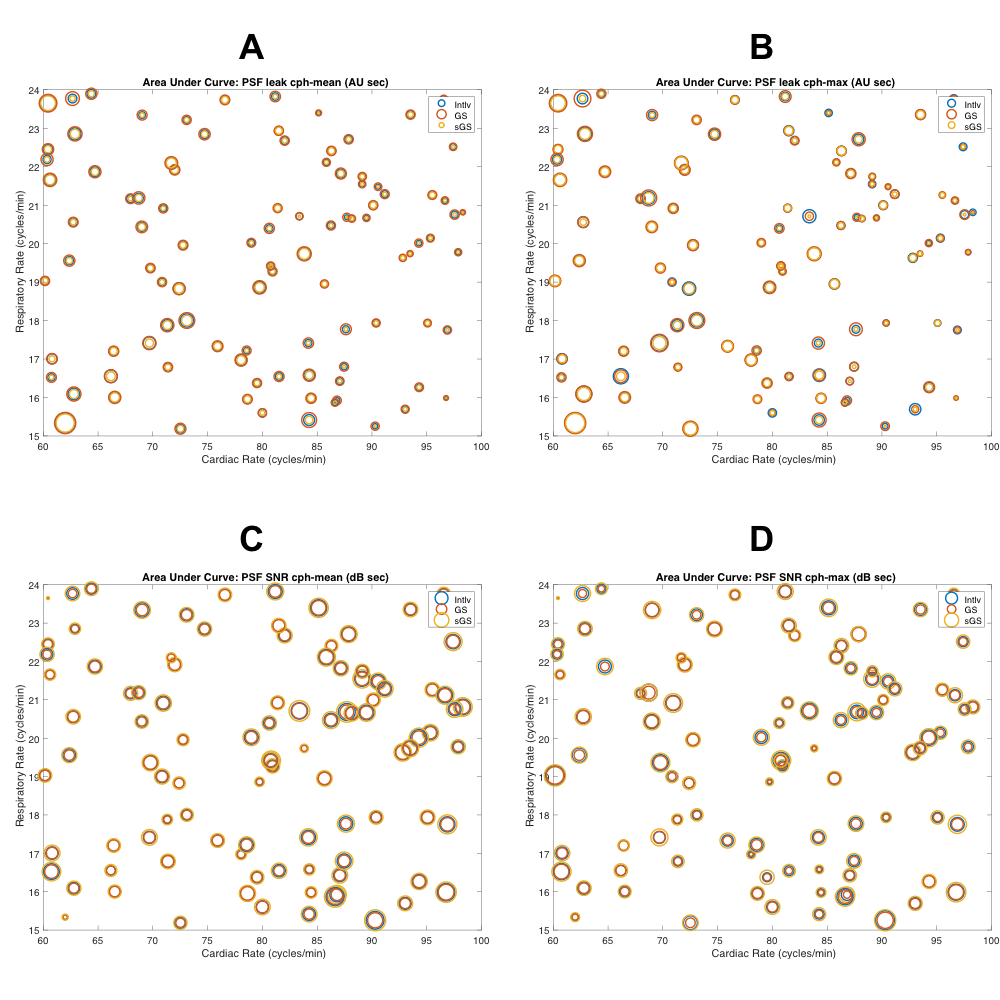


Figure 4. Area-under-curve (AUC) metric values of all 100 simulated cardiorespiratory self-gated scans plotted as scatter plots with randomly generated cardiac and respiratory rates on the *x*- and *y*-axes. AUC values are encoded in marker size. Note that at all motion rates, the sGS’ PSF leak (lower is better) is consistently the lowest among the three PE schemes (yellow circles are always innermost), for both cardiac-phase mean (A) and max (B). For PSF SNR (higher is better), the sGS is consistently the greatest among the three (yellow circles are always outermost), for both cardiac-phase mean (C) and max (D).

# References

1. Guo L, Derbyshire JA, Herzka DA. Pseudo-projection-driven, self-gated cardiac cine imaging using cartesian golden step phase encoding. Magn Reson Med. 2016;76:417–29.

2. Tsao J, Kozerke S, Boesiger P, Pruessmann KP. Optimizing spatiotemporal sampling for k-t BLAST and k-t SENSE: application to high-resolution real-time cardiac steady-state free precession. Magn Reson Med. 2005;53:1372–82.

3. Candes EJ, Romberg J, Tao T. Robust uncertainty principles: exact signal reconstruction from highly incomplete frequency information. IEEE Trans Inf Theory. 2006;52:489–509.

4. Lustig M, Donoho DL, Santos JM, Pauly JM. Compressed Sensing MRI. IEEE Signal Process Mag. 2008;25:72–82.

5. Feng L, Grimm R, Block KT, Chandarana H, Kim S, Xu J, et al. Golden-angle radial sparse parallel MRI: Combination of compressed sensing, parallel imaging, and golden-angle radial sampling for fast and flexible dynamic volumetric MRI. Magn Reson Med. 2014;72:707–17.
